# Was an aortic valve replacement with a mechanical valve the right option all along? Cogan’s syndrome with recurrent aortic regurgitation: a case of evolving surgical decisions

**DOI:** 10.1093/omcr/omaf143

**Published:** 2025-08-20

**Authors:** Maram Gad, Lanya Faiq, Emma Arifagic, Alex Kamougeros, Stelios Ioannou, George Shiakos, Ioannis Tzanavaros

**Affiliations:** European University Cyprus, School of Medicine, 6 Diogenous Street, Nicosia, Engomi 2404, Cyprus; European University Cyprus, School of Medicine, 6 Diogenous Street, Nicosia, Engomi 2404, Cyprus; European University Cyprus, School of Medicine, 6 Diogenous Street, Nicosia, Engomi 2404, Cyprus; Apollonion Private Hospital, Cardiac Innovation Center, Nicosia, Cyprus; Adult and Pediatric Cardiac Surgery Department, Cardiac Innovation Center of Apollonion Private Hospital, Nicosia, Cyprus; European University Cyprus, School of Medicine, 6 Diogenous Street, Nicosia, Engomi 2404, Cyprus; Adult and Pediatric Cardiac Surgery Department, Cardiac Innovation Center of Apollonion Private Hospital, Nicosia, Cyprus; Adult and Pediatric Cardiac Surgery Department, Cardiac Innovation Center of Apollonion Private Hospital, Nicosia, Cyprus; University of Nicosia Medical School, School of Medicine, Nicosia, Cyprus

**Keywords:** cardiology and cardiovascular systems, rheumatology, Audiovestibular medicine, immunology, medical ophthalmology

## Abstract

Cogan Syndrome (CS) is a rare autoimmune disease, complicated by a variety of cardiac manifestations. This case report represents the only documented case in the Republic of Cyprus. It describes the experience of a 23- year-old woman with CS who presented with newly diagnosed, severe aortic regurgitation (AR) and suspected endocarditis. The patient was initially treated successfully with a Ross procedure, but the recurrence of acute AR a few years later, led to the decision to treat her with a mechanical valve replacement, minimising complications and providing as permanent of a surgical solution as possible. This case demonstrates the importance of individualising treatment for such patients

## Introduction

Cogan Syndrome (CS) is an autoimmune, multisystemic disease marked frequently by intraocular inflammation, vestibulo-auditory dysfunction, and interstitial keratitis, treated with corticosteroids [1]. Upper respiratory infection is suspected as a cause but lacks scientific validation, with only 250 documented cases worldwide [2]. Systemic vasculitis, affecting medium and large vessels, is the main pathomechanism in 15%–21% of cases [2]. As the sole symptom, vasculitis can delay diagnosis, leading to life threatening aortic complications [1]. Aortitis is commonly reported, causing cusp degeneration, thickening, deformation, or prolapse, and 10% of patients develop severe complications like aneurysms or aortic insufficiency, often presenting as AR [2, 3].

## Case report

**Figure 1:**
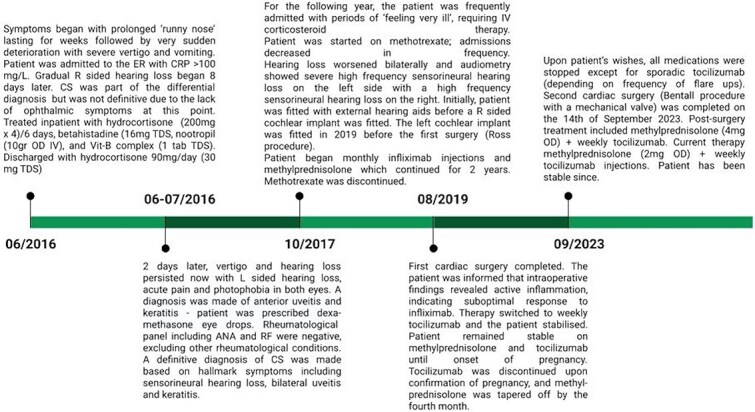
Timeline of disease progression, diagnosis and landmark treatment interventions until present date.

This is the case of a 23-year-old female with CS (Fig. 1) who was diagnosed with AR in March 2019. She complained of mild fatigue and dyspnoea and a transthoracic echocardiography (TTE) revealed mild–moderate aortic valve regurgitation, most likely as a vasculitic manifestation of CS (Table 1). She returned in early July 2019, with sudden onset of severe fatigue, dyspnoea and orthopnoea. A TTE was done during this acute presentation which revealed progressed, severe AR, with preserved left ventricular ejection fraction (60%–65%) and normal dimensions. Due to the severity of the diagnosis and presentation, the patient was urgently transferred to Germany in July 2019, where she was admitted with signs of cardiac decompensation. Her medical history included recent cochlear implants and no prior heart disease. At the time, the patient was on Prednisolone (20 mg daily), Leflunomide (20 mg once daily), and Tocilizumab (162 mg weekly). Urgent surgical therapy was indicated and during surgical planning, the patient expressed her wish to have children. The preferred approach of mechanical valve replacement utilized normally in this age group was therefore excluded, due to the inherent thrombogenicity of mechanical prostheses and the hypercoagulability of pregnancy, compounding the risk of thrombotic complications [4]. Alternatively, the patient was offered a Ross procedure, which replaces the aortic valve with a pulmonary autograft and the pulmonary valve with a donor valve, offering a safer alternative during pregnancy and excellent long-term outcomes [5].

**Table 1:** Available immunologic and inflammatory values at various points of diagnosis, instances of flare up and treatment.

**Lab Values**	**Lab Values** **(Normal Range)**	**Before Diagnosis** **of CS (06/2016)**	**After Diagnosis** **of CS (08/2016)**	**First AR Diagnosis (03/2019)**	**Second AR Diagnosis (14/09/2023)**
ANA	<1:80	Negative	-	-	-
RF	<20 IU/mL	Negative	-	-	-
WBC (U/L)	(4.37–9.68 10^9/L)	6.61	10.2	9.54	11.6
Hb (gr/dL)	Female (10.6–13.5)	14.4	13.4	11.5	13.3
ESR (mm/1h)	(0–20)		-	65	-
CRP (mg/L)	(0.00–5.00)	150	0.16	119.3	3.44

The procedure was performed in late July 2019. Intraoperatively, the aortic valve appeared completely degenerated, where only the left coronary cusp was anatomically intact. The aortic wall showed macroscopic signs of acute and chronic inflammation transitioning to healthy tissue at the level of the distal ascending aorta. The resected valve was sent for microbiological culture with suspected acute, fulminant bacterial endocarditis, which was subsequently positive for *Staphylococcus epidermidis*. The patient was therefore treated with Ampicillin, Flucloxacillin, Gentamicin, and Vancomycin for 4 weeks. Despite no fever or positive blood cultures, endocarditis was considered certain at the time due to the macroscopic appearance of the valve and the presence of *S. epidermidis* in the cultured sample and thus treated as such. After the successful surgery, the patient delivered a healthy child and remained asymptomatic. The patient wished to discontinue all medications remaining on sporadic Tocilizumab therapy (Fig. 1) under her rheumatologist’s supervision.

**Figure 2:**
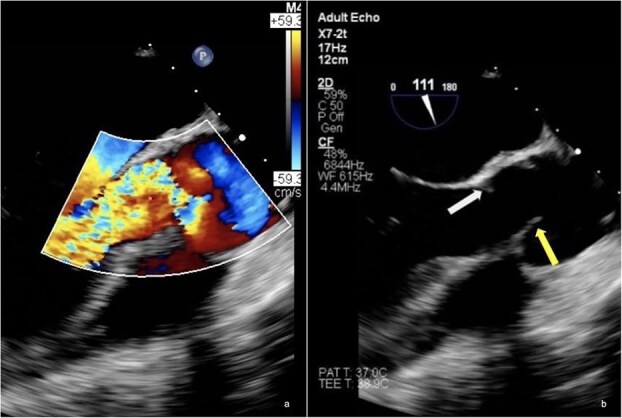
(A) TOE Doppler of aortic valve exhibiting severe degree of aortic regurgitation. (B) Mid-oesophageal aortic valve long axis view with doppler. Absence of coaptation between non-coronary and right-coronary cusps, indicating, most likely, a mechanical failure tear in the NCC/RCC commissural region. White arrow—Absent NCC, yellow arrow—Normal RCC.

In early 2023, the patient returned with severe fatigue and dyspnoea, similarly to her first episode in 2019*.* The patient described an acute onset of symptoms; had rapid clinical deterioration and no episodes of fever or recent infections. On auscultation, a characteristic early diastolic murmur was heard. A diagnosis of recurrence of severe AR was confirmed by TTE and a transoesophageal echocardiography (TOE) was done highlighting the severity of the AR with complete absence of cusp coaptation, possibly due to tear of the RCC (Fig. 2a). A secondary, moderate mitral valve regurgitation was also detected, likely a result of the severe increase in aortic volume due to the AR.

Surgery was indicated, and in September 2023, the patient underwent a mechanical Bentall procedure using a 21 mm valve conduit. This time, a mechanical valve was chosen as the patient did not plan on having any more children. A large tear in the RCC was found, beginning at the highest point of the RCC/NCC commissure (Fig. 2b). Macroscopically, the valve and the neoaortic tissue demonstrated evidence of inflammation, as in her previous surgery. Samples were sent for evaluation and histology confirmed marked inflammation with necrotic cell debris, affecting the valve cusps. As a precaution, the patient was placed on triple antibiotic therapy (Vancomycin, Gentamicin and Rifampicin) but discontinued once the blood and intraoperative cultures returned negative—ruling out endocarditis entirely. The patient recovered quickly and was discharged 10 days postoperatively. INR levels were strictly monitored by her cardiologist as per routine testing for Warfarin therapy. Cortisone therapy (2 mg OD) and weekly tocilizumab were resumed by her Rheumatologist and her 7 and 30-day postoperative follow-ups were uneventful.

## Discussion

This case highlights the complex and progressive nature of aortic valve pathology in CS, emphasizing the interplay between autoimmune vasculitis and surgical intervention. It underscores the challenges in distinguishing vasculitic damage from infectious causes and in selecting appropriate long-term management strategies.

The initial presentation of AR in March 2019, followed by rapid progression to severe AR in July 2019, underscores the aggressive cardiac manifestations of CS. The Ross procedure, chosen to preserve the patient’s reproductive potential, initially provided symptom relief. However, recurrent AR in 2023, accompanied by histological evidence of inflammation and necrosis in the valve and neoaortic tissue, reaffirmed that vasculitis was the primary driver of valve degeneration, not infection. The equivocal identification of *S. epidermidis* in valve cultures during the initial surgery likely represented contamination, consistent with the literature, where coagulase-negative staphylococci are frequent contaminants in prosthetic valve cases [6].

Preoperative CT angiography performed prior to the second surgery in 2023 revealed no evidence of coronary artery disease or pathology involving the ascending aorta, confirming that the patient’s severe AR and its associated sequelae were localized to the valvular structures. This finding further supported the hypothesis that systemic vasculitis, rather than structural or atherosclerotic abnormalities, was the underlying cause of the patient’s recurrent valve pathology.

Importantly, this case underscores the limited durability of biological grafts in the setting of uncontrolled systemic vasculitis. In a similar case, a 19 year old female patient had severe aortic insufficiency and underwent aortic valve replacement with a biological prosthesis, due to her desire to have children [7]. While the case highlights the need for a biological valve to avoid the use of anticoagulation, our case proves this was suboptimal, necessitating the use of a mechanical valve in the patient’s case. Likewise, another report describes a case of aortic regurgitation in a 35 year old male treated with a mechanical valve replacement due to its longevity and favorable profile suited for the patient’s age and prosthesis durability, with very favorable results [8]. Nevertheless, current literature urges the need to promptly correct AR in CS to achieve a better prognosis. The Ross procedure, while an ideal option for patients desiring pregnancy, failed under the inflammatory burden of CS. This failure raises important questions about whether prolonged or more aggressive immunosuppressive therapy could have mitigated the recurrence of AR and improved graft longevity.

The successful transition to a mechanical Bentall procedure in September 2023, after the patient’s decision to forgo further pregnancies, highlights the long-term durability and efficacy of mechanical valves in managing severe AR in CS. The lack of any infectious evidence during the second surgery further confirmed that the underlying pathology was autoimmune.

This case underscores the critical need for a multidisciplinary approach in managing CS, combining rheumatologic expertise to optimize immunosuppressive therapy and cardiothoracic intervention to address progressive valve pathology. It also emphasizes the importance of individualized treatment planning, taking into account patient-specific goals, such as future pregnancy, while addressing the aggressive vasculitic damage inherent in CS.

Further research is needed to explore the role of advanced immunosuppressive regimens in preventing valve degeneration in CS, as well as the comparative outcomes of biological versus mechanical valve replacements in this population. This case suggests that, in the absence of pregnancy plans, mechanical valve replacement offers a definitive and durable solution to recurrent AR caused by CS-associated vasculitis.
